# Colonic Atresia due to Internal Herniation through the Falciform Ligament Defect: A Case Report

**Published:** 2014-04-01

**Authors:** Varsha Soni, Prakash D. Valse, Sameer Vyas

**Affiliations:** 1 Department of Pediatric Surgery, J.L.N. Medical College, Ajmer; 2 Department of Surgical Gastroenterology, J.L.N. Medical College, Ajmer; 3 Department of General Surgery, J.L.N. Medical College, Ajmer

**Keywords:** Colonic atresia, Internal herniation, Falciform ligament

## Abstract

Colonic atresia is the rarest outcome of all gastrointestinal type of internal hernia. We report a case of neonate with atresia of the transverse colon caused by herniation of the transverse colon through a defect in falciform-ligament.

## INTRODUCTION

Colonic atresia is a rare form of intestinal atresia.[1] Internal herniation secondary to non-peritonealization of falciform-ligament accounts for 0.2% of internal hernia. Age may range from neonate to 92 years.[2] Small bowel herniation is most common. We report a case of atresia of the transverse-colon due to internal herniation through the falciform ligament in a neonate.

## CASE REPORT

A 3-day-old full term baby girl, weighing 2.48 kg, developed progressive abdominal distension, bilious vomiting, and failure to pass meconium since birth. Anal opening was normal. Plain roentgenogram revealed distal intestinal obstruction. Patient was optimized for operation. At operation, there was a grossly dilated segment of transverse colon that had herniated through a defect in the falciform ligament(Fig.1).Further exploration and mobilization of falciform ligament revealed a type III atresia of the transverse-colon(Fig. 2). Both ends of the colon were exteriorized. Distal micro-colon was examined for its patency till anus. Serial biopsies were taken to rule out Hirschsprung’s disease. Postoperatively patient was kept on broad-spectrum antibiotics and antifungal (fuconazole 6mg/kg i.v.o.d.) as the blood culture showed budding yeast cells. Histopathology report ruled out Hirschsprung’s disease. Patient was discharged on 10th postoperative day.Postoperatively, the patient developed frequent colostomy prolapses which were dealt by re-position of prolapsed bowel followed by use of a soft silicone nipple of feeding bottle, with wide aperture, for collection of evacuations inside it. This made cleaning of patient easy and also prevented excoriations. At three and half months of age, colostomy was reversed. The patient was discharged in a good condition. 

**Figure:**
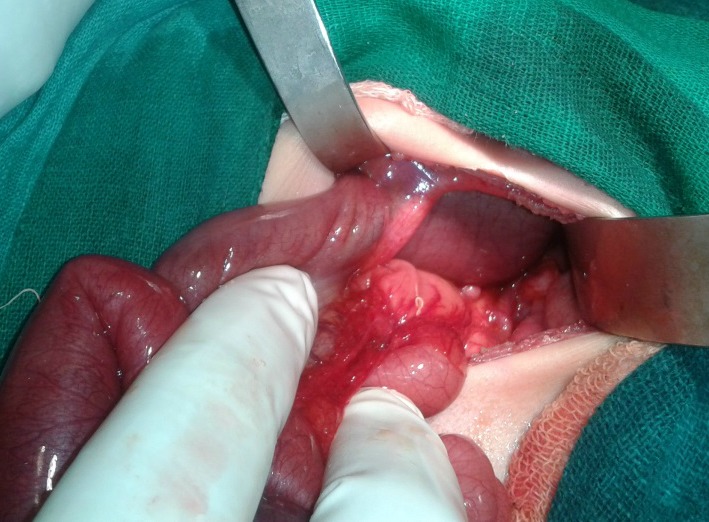
Figure 1: Dilated transverse colon herniating through falciform ligament.

**Figure:**
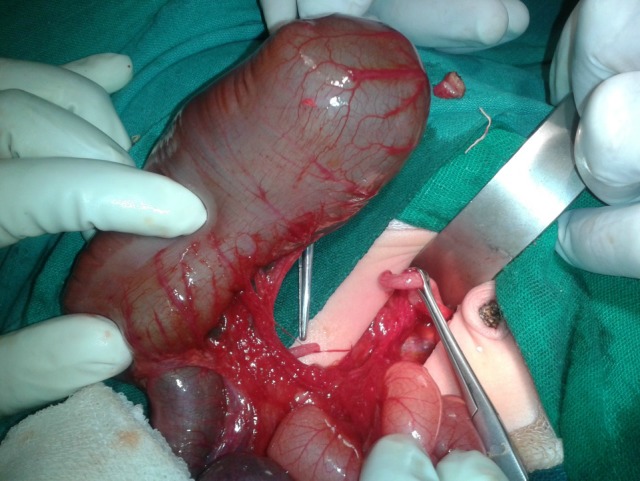
Figure 2: Transverse colonic atresia after ligation of umbilical vein.

## DISCUSSION

Atresia is a sequel of in-utero vascular insult which may result secondary to intussusception, volvulus, thrombo-embolic events, incarceration or strangulation secondary to internal herniation, and abdominal wall defects. Internal herniation through falciform ligament is a rare type of internal hernia. The first reported case of falciform-ligament internal hernia was described by Schultz and Ziegler in 1937.[2] A literature search revealed about 45 cases of falciform ligament internal hernia;only six were reported in neonates.[2-9] Two neonates had antral obstruction,[4] one had ileal atresia due to mid ileal entrapment in defect of falciform ligament;[6] rest of the neonates had small bowel obstruction or strangulation. Our case is the seventh one and the only case where internal herniation through the falciform ligament resulted in colonic atresia. On exploration, the umbilical vein needs to be sacrificed to release the herniated content. Bowel resection may be required in 43% of the cases with a mortality of 12%.[9] To conclude, our case illustrated an important etiology of vascular insult leading to transverse colon atresia.

## Footnotes

**Source of Support:** Nil

**Conflict of Interest:** None

